# A concise overview of using the P300 component of the event-related potential for objective estimation of visual acuity

**DOI:** 10.3389/fopht.2026.1846725

**Published:** 2026-06-26

**Authors:** Julia Haldina, Michael Bach, Céline Z. Duval, Michael B. Hoffmann, Sven P. Heinrich

**Affiliations:** 1 Eye Center, Medical Center – University of Freiburg, Faculty of Medicine, University of Freiburg, Freiburg, Germany; 2 Faculty of Biology, University of Freiburg, Freiburg, Germany; 3 Department of Ophthalmology, University Hospital, Otto-von-Guericke University, Magdeburg, Germany; 4 Center for Behavioral Brain Sciences, Magdeburg, Germany

**Keywords:** event-related potential, human, objective acuity estimation, P300, visual acuity

## Abstract

Visual acuity (VA) assessment traditionally relies on patient cooperation, which can be unreliable in pediatric cases, cognitive impairment, or suspected malingering. While the visual evoked potential (VEP) provides an objective alternative, it primarily reflects the integrity of the early visual pathways and may overestimate VA in conditions like amblyopia. This concise review explores the P300 event-related potential as a promising cognitively-driven tool for objective VA estimation. A literature search was conducted to identify studies utilizing the P300—a marker of high-level cognitive processing and conscious perception—for VA estimation. Additional literature was included to provide a broader context. Unlike the VEP, which depends on physical stimulus characteristics, the P300 is elicited during conscious stimulus categorization, typically using an oddball paradigm. Therefore, P300-based VA estimation can utilize standard clinical optotypes, such as Landolt Cs, allowing for a more direct comparison with gold-standard psychophysical testing. For instance, P300-based VA mirrors psychophysical deficits in amblyopia better than VEP-based methods. The P300 serves as a valuable objective complement to traditional visual electrophysiology, bridging the gap between physiological stimulus processing and conscious perception. While challenges such as recording duration still need to be resolved, the ability to use custom-tailored stimuli offers significant potential for forensic evaluations and complex clinical cases in ophthalmology.

## Introduction

Visual acuity (VA) is traditionally a “subjective” psychophysical measure relying on patient cooperation. However, in cases of cognitive impairment, pediatric care, or situations of suspected malingering, patient responses are often unreliable or manipulated (1–3). Misrepresentation in VA testing is also a challenge in vision-impaired sports (4). Conventional electrophysiological alternatives, primarily the visual evoked potential (VEP), have been successfully applied in many of these cases (5). By measuring responses to differentiatingly coarse or fine checkerboard patterns or gratings, they provide objective data but face two main challenges:

1. They are limited to measuring the integrity of the eyes and the pathways leading towards the primary visual cortex (V1), but are insensitive to visual impairments of cortical origin if some residual response is evoked in V1.
2. If a visual impairment is caused by image distortion or fragmentation, the conversion of a VEP-based measure into a VA value that corresponds to standard optotype VA is not reliable (6). This is because a distorted and fragmented checkerboard can still be a good VEP stimulus, while the same type of degradation might render optotypes impossible to read.

The relevance of the second point arises from the fact that psychophysical optotype VA is the accepted gold standard, familiar to clinicians and used as a reference to validate VEP-based methods. It would be incorrect to say that VEP-based VA is erroneous merely because it has different characteristics. Rather, these differences may provide meaningful insights into the underlying pathologies.

Like the VEP, the P300 is an event-related potential (ERP) component and part of the electroencephalogram (EEG). However, in contrast to the VEP, which is “evoked” (meaning that it depends directly on the physical stimulus characteristics), the P300 reflects high-level cognitive processing. It is associated with conscious stimulus categorization and perception (7, 8). As such, as discussed below, it constitutes a valuable complement to the VEP and holds promise for being particularly useful in the aforementioned challenging cases.

## Literature search

As a starting point for the present review, we performed a Pubmed search using the query “*visual acuity” AND (“P300” OR “P3” OR “P3b”)* (search date February 27^th^, 2026). This search identified 153 hits, whose titles and/or abstracts were screened for relevance to P300-based estimation of VA or confirmation of reduced VA. We retained 10 articles that were within this scope and written in English (9–18), summarized in **Table 1**. Additional literature served to narratively embed P300-based VA testing into a broader context.

**Table 1:** Main references.

Authors (year)	Ref.	Study characteristics	Main findings
Towle et al. (1985)	(9)	Three patients, clinical visual deficits, grating stimuli, orientation oddball. Two spatial frequencies above and below claimed perceptual threshold compared. Healthy controls with different tasks.	P300 responses were also obtained in response to stimuli that the patients claimed not to see. In controls, P300 was largest when stimuli were task-related. **Study provides basic proof of concept.**
Heinrich et al. (2010)	(10)	Twelve healthy participants, grating oddballs among homogenous gray stimuli. Four spatial frequencies, artificial dioptric blur.	Without blur, similar P300 responses obtained for all spatial frequencies. With blur, P300 was absent below the recognition threshold, with a rather sharp transition between seen and unseen stimuli. **Study lays foundation for P300-based acuity estimation.**
Jiraskova et al. (2011)	(11)	Case study, “functional” loss of vision following chemical eye burn, VEP normal. 1-year follow-up, P300 recorded to letters and oddball digits.	P300 initially absent; both vision and P300 recovered without treatment after one year. **Study supports link between P300 and subjective vision.**
Heinrich et al. (2015)	(12)	Twelve healthy participants, Landolt Cs as oddballs among frequent closed rings. Three levels of artificial dioptric blur, 6 stimulus sizes or spatial frequencies, respectively. For comparison, 12 further participants tested with grating stimuli similar to those used by Heinrich (10).	P300 amplitude shows a sigmoid trajectory when optotype visibility transitions from unrecognizable to recognizable. **Study demonstrates feasibility of using optotype and demonstrates a sigmoid relationship resembling a psychometric function.**
Marhöfer et al. (2015)	(13)	Eleven healthy participants; high-pass filtered unscrambled self-facial images as oddballs among frequent scrambled images. Artificial dioptric blur.	P300 reflects recognizability of faces. **Study demonstrates that filtered stimuli other than gratings and optotypes may be used in P300-based acuity estimation.**
Beusterien & Heinrich (2018)	(14)	29 healthy participants, Landolt C optotypes as oddballs among frequent closed rings. Artificial blur created either by a frosted diffuser pane or by a patterned pane placed in front of monitor, both adjusted to yield similar psychophysical acuity.	Similar results obtained for both types of stimulus degradation, in contrast to VEP outcome in earlier study. **Study suggests P300 approach to be more closely related to standard psychophysical acuity than are VEP-based methods, particularly with respect to disease-specific effects.**
Huang et al. (2018)	(15)	Twenty-two healthy participants, pairs of stimuli (tumbling-E style optotypes, matching or different orientations) in three sizes were used instead of oddball sequence.	P300 was elicited by supra-threshold optotypes. **Study extends previous studies by showing that a paired-stimulus paradigm offers an alternative to oddball sequences.**
Jia et al. (2023)	(16)	Thirty-two participants, uncorrected myopia. Oddball sequence made up from tumbling E optotypes, one standard orientation and one oddball orientation. Oddballs varied in size.	Similar P300 responses were recorded for all sizes. Confoundingly, the oddball stimuli were not only defined by orientation but also by size. **Study confirms that optotypes can be used to elicit a P300, although the study is not straightforwardly interpretable in terms of acuity estimation.**
Duval et al. (2024)	(17)	Twelve healthy participants, two types of oddball stimuli (one with the critical detail in the center, dubbed “FreiBurger”) were compared. Vision was strongly artificially degraded, with and without visual field constriction. Single large optotype size tested, otherwise similar paradigm as in Heinrich (12).	With a constricted visual field, no reliable P300 was recorded with Landolt Cs despite optotypes being large enough for acuity level. With FreiBurger optotypes, P300 responses could be recorded under otherwise identical conditions. Comparability of Landolt C and FreiBurger optotypes additionally confirmed psychophysically. **Study suggests that FreiBurger optotypes are suitable for patients with a constricted visual field combined with low acuity.**
Gopiswaminathan et al. (2024)	(18)	Eighteen participants with amblyopia, FreiBurger optotypes, fast stimulus sequence (31), otherwise similar to Heinrich (12).	In contrast to VEP-based acuity estimates, the P300-based approach is not invalidated by amblyopia. The study estimates a conversion parameter that relates raw P300-based threshold to psychophysical visual acuity, irrespective of amblyopia. **Study demonstrates that P300-based acuity estimation in amblyopia is quantitatively similar to the non-amblyopic case and specifies the relationship to psychophysical acuity.**

## Basic characteristics of the P300

**Figure 1:**
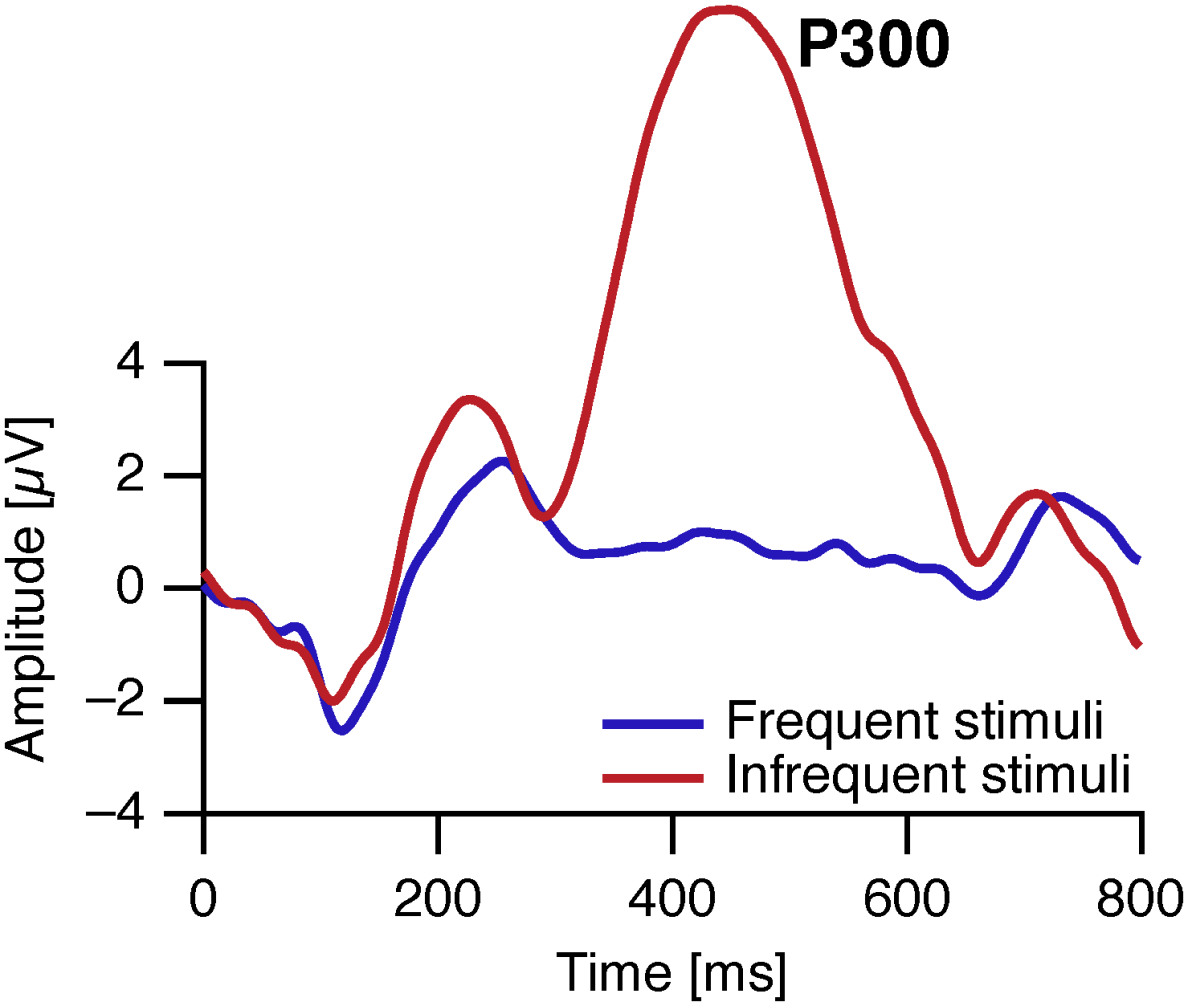
Example of a P300 response elicited by infrequent stimuli (red trace) in comparison to the response to frequent stimuli (blue); data replotted from (21). Computing the difference between both responses yields a net P300, the amplitude of which may feed into further analysis (e.g., **Figure 2**).

The P300 is an ERP marker of high-level cognitive processing, independent of the sensory domain, that has been associated with conscious perception (19). It was initially described by Sutton et al. (20). Most commonly, the P300 is measured at parietal locations using an oddball paradigm, where frequent stimuli of one type are interspersed with infrequent stimuli of another type. The P300 is elicited by these infrequent stimuli and occurs typically later than 300 ms after stimulus onset (**Figure 1**). The precise processes reflected by the P300 are not yet fully understood (22). It is clear, however, that the P300 consists of several subcomponents, including the P3b, which is often implied when the term “P300” is used. The present review follows this tradition.

Importantly, recording the P300 is not limited to gratings, checkerboards, or similarly massive visual stimuli. Rather, an oddball sequence can be created from any stimuli, as long as they are immediately distinguishable.

## Early history of P300 application in vision impairments

As early as 1985, Towle et al. (9) reported on the use of the P300 in three patients with clinically diagnosed functional visual deficits, concluding that the P300 “can be helpful in assessing the subjective visual experience of patients suspected of having functional visual loss”. In a different sensory modality, specifically the somatosensory domain, Lorenz et al. (23) performed a broadly similar study and were able to differentiate between malingering and conversive sensory loss. The general idea was picked up by Heinrich et al. (10) in 2010, who demonstrated that P300-based VA estimates can be obtained by using grating stimuli with varying spatial frequencies. A report from 2011 by Jiraskova et al. (11) describes a case of visual loss where all physiological correlates of visual function, including VEPs, were unremarkable except for the absence of the P300, supporting the notion that the P300 can be useful in the investigation of unexplained visual loss. P300-based VA estimation might be considered conceptually related to P300-based detection of concealed information in incriminatory investigations (24). There, it is not visual recognizability of a stimulus attribute that is probed, but the recognition of relatedness to a crime.

Leveraging the fact that the P300 does not require a massive visual stimulus to be elicited, Heinrich et al. (12) demonstrated that Landolt C optotype stimuli can be used to create an oddball sequence to obtain P300-based estimates of VA. This means that the same stimuli can be used for P300 recordings as for conventional psychophysical chart-based testing. Without reference to earlier studies, Jia et al. (16) provided further evidence that the P300 can be recorded using optotype stimuli.

## Specific applications

### Amblyopia

Because they share similarities with psychophysical testing, P300-based techniques provide a perspective to mitigate problems inherent in VEP-based estimation, including the overestimation of VA in amblyopia (25), which is likely related to the VEP-based approach being relatively robust against stimulus distortions and impairment of cortical processing. This expectation is supported by Beusterien and Heinrich (14), who have demonstrated that psychophysical VA and P300-based estimates are similarly affected by experimentally induced stimulus distortion and fragmentation provided that both employ optotypes. More recently, Gopiswaminathan et al. (18) confirmed this assumption by demonstrating in patients that the amblyopia-related reduction of psychophysical optotype VA is in fact paralleled by P300-based estimates.

### Other conditions

Several studies have demonstrated that cerebral visual impairment (CVI) is associated with altered P300 responses (26). Although CVI could be considered a prime candidate for the usefulness of P300-based VA testing, especially as it bypasses the need for motor or verbal responses, this specific application has, to our knowledge, not yet been investigated. Actually, the case of unexplained transient visual loss with selectively abolished P300 reported by Jiraskova et al. (11) might have had cerebral origins. The theoretical alignment between the cognitively mediated nature of the P300 and the specific challenges of CVI suggest significant clinical potential for this objective approach.

P300-based VA testing using optotypes is likely to perform well in macular conditions, where pattern-based techniques such as VEP measurements may show poor agreement with psychophysical VA (5). Here again, specific evidence is lacking.

### Children

Although children are a primary target group, P300-based VA estimation has not yet been tested in this population. While response characteristics may differ in an age-dependent manner from the adult P300, it seems highly plausible that P300-based VA estimation is feasible, provided that the particular needs and abilities of the respective age group are appropriately addressed. This assumption is also supported by studies that used very similar techniques to investigate specific aspects of face and object perception in healthy children (27), as well as brain-computer interface approaches as an alternative communication tool in children with severe physical impairments (28).

## Linking the P300 to psychophysics

**Figure 2:**
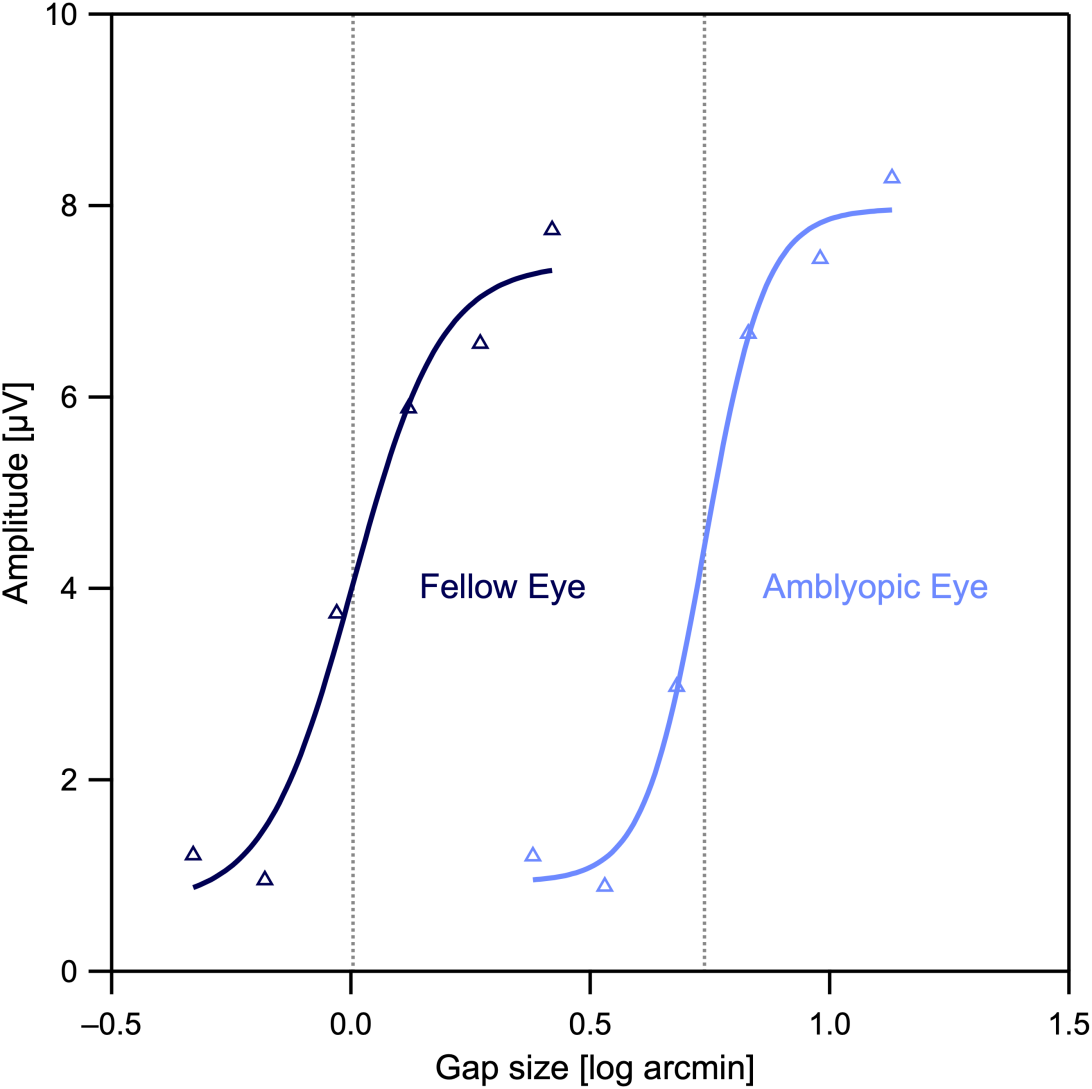
P300 amplitudes recorded to oddball optotypes in amblyopic patients. Each marker represents an optotype size. The right group of markers corresponds to the amblyopic eye, and the left group to the fellow eye. Cumulative Gaussians were fitted to the respective data points. [Data replotted from (18)].

Estimating VA via the cognitive P300 component shares significant similarities with psychophysical testing, as both methods necessitate higher-order cognitive processing. This is also reflected by the finding that the dependence of the P300 amplitude on stimulus size or coarseness can, in analogy to psychophysical stimulus dependence, be well described by a sigmoid function (12, 18), as depicted in **Figure 2**. By incorporating both suprathreshold and subthreshold stimuli, the analysis functions as an intra-individual comparison, which reduces sensitivity to inter-individual differences, e.g. in P300 amplitude.

One might expect both approaches, psychophysical and P300-based, to yield the same threshold, as both rely on the recognition of the stimuli. However, this is not the case, even when both methods use the same optotypes (12, 14). Therefore, a conversion parameter is required if one wants to use a P300-based threshold to estimate the corresponding psychophysical VA.

An explanation for the conversion parameter likely involves the temporal constraints of optotype recognition. Generating a robust P300 response requires successful stimulus identification within the initial 100–200 ms of presentation. This contrasts sharply with conventional psychophysical VA assessments, where observers usually have unlimited time to inspect the targets. Research indicates that such abbreviated exposure times negatively impact VA estimates; for example, in a psychophysical study Heinrich et al. (29) demonstrated a 0.29 logMAR discrepancy when reducing presentation duration from 1.0 s to 0.1 s. While conversion parameters are likely contingent on stimulation parameters, the results of Gopiswaminathan et al. (18), who compared amblyopic and fellow eyes, suggest a wide applicability across diseases. A broader investigation remains to be conducted.

The P300 is extracted from the EEG by means of stimulus-synchronized averaging. Assuming that P300 generation is mediated by stimulus perception, this approach tacitly requires that the moment of perception occurs in a sufficiently reproducible manner relative to stimulus onset. Meeting these temporal requirements becomes particularly challenging in patients with low VA and with visual field defects. They might initially miss the critical detail of an optotype if it changes location between trials, as is the case with large Landolt C optotypes. Searching for the critical detail introduces large temporal variability and, if associated with eye movements, additional signal artifacts. In a recent study, Duval et al. (17) achieved a substantial improvement in this respect by using novel optotypes (dubbed “FreiBurger”) that feature the critical detail in the center irrespective of their orientation or size.

## Challenges in P300-based VA estimation

### Attention

An important point is the P300’s susceptibility to the diversion of attention. While this sensitivity might be seen as a key benefit, allowing for the detection of attention-related visual deficits, it may potentially render the technique vulnerable to malingering if attention is intentionally withheld. Although preliminary data illustrate this challenge, they also suggest that the technique is sufficiently robust (30). Resistance to malingering might be further enhanced by utilizing alternative stimuli. For instance, Marhöfer et al. (13) demonstrated that P300-based VA estimates may be obtained with high-pass filtered self-facial images, building on their previous finding that face stimuli are relatively resilient against attentional diversion (31). In a study using matching and non-matching stimulus pairs above and below the VA threshold, Huang et al. (15) found the visual P300 to be robust against auditory distraction. Beyond malingering, attention is also a pertinent factor during lengthy recording procedures, particularly in pediatric or cognitively impaired populations, who might be particularly susceptible to fatigue.

### Recording duration

P300 recordings traditionally require relatively long recording durations. This is due to the need for multiple frequent stimuli for each infrequent target to elicit a sizable response (21), combined with the P300’s late occurrence and broad peak shape, which typically necessitate trial lengths of around 800–1000 ms. However, this may be ameliorated by using fast (e.g., 200 ms between stimulus onsets) randomized stimulus sequences, which allow for deconvolution of temporally overlapping responses (32).

Another option is so-called “frequency-tagging”, where infrequent stimuli are presented at regular intervals, such that an oddball response manifests in the recorded signal at the respective frequency and its higher harmonics (33). In this approach, the inter-stimulus intervals can also be very short, and due to the periodicity of stimulation, analysis in the frequency domain allows for efficient response detection and quantification. However, this approach precludes easy disentanglement of P300-related response components and other response differences between frequent and infrequent stimuli.

The aim of reducing examination times might also be achieved by optimizing the testing strategy. For instance, rather than testing a wide range of stimulus sizes, it may be sufficient to present only a single, strategically chosen size that yields the decisive information for the clinical or forensic question at hand. While conventional slow oddball paradigms would require approximately 10 minutes of net recording time, fast-sequence paradigms can reduce this to approximately 3 minutes.

## Perspectives

A major benefit of P300-based testing is the flexibility to use custom-tailored stimuli for specific applications, such as the investigation of crowding, specific visual field locations, or more complex perceptual tasks. As long as frequent and infrequent stimuli can be swiftly discriminated, there are few limitations to the stimulus design. First steps with non-standard stimuli have been undertaken by Marhöfer et al. (13), who used face images, and by Nikolaidou and Strasser (34), who combined the Panda Illusion with P300 recordings to estimate VA.

The VEP reflects primarily stimulus processing in early visual cortex, and the P300 is related to high-level cognitive processes and possibly conscious perception. Consequently, it remains an obvious target for research to address intermediate levels along the processing chain using associated ERPs. Assessing their utility as markers of stimulus processing could identify complementary candidates for objective visual function testing.
